# A pilot study: handgrip as a predictor in the disease progression of SCA3

**DOI:** 10.1186/s13023-023-02948-3

**Published:** 2023-10-11

**Authors:** Chungmin Chiu, Wenling Cheng, Yongshiou Lin, Tatsung Lin, Huiju Chang, Yujun Chang, Chiaju Lee, Henhong Chang, Chinsan Liu

**Affiliations:** 1 Graduate Institute of Integrated Medicine, College of Chinese Medicine, China Medical University, Taichung, Taiwan; 2 Department of Chinese Medicine, Changhua Christian Hospital, Changhua, Taiwan; 3 Vascular and Genomic Center, Institute of ATP, Changhua Christian Hospital, Changhua, Taiwan; 4 Center of Regenerative Medicine and Tissue Repair, Institute of ATP, Changhua Christian Hospital, Changhua, Taiwan; 5 Big Data Center, Changhua Christian Hospital, Changhua, Taiwan; 6 Department of Neurology, Changhua Christian Hospital, 7F., No.235, Syuguang Rd., Changhua, 500 Taiwan; 7 Department of Chinese Medicine, China Medical University Hospital, No.91, Xueshi Rd., North District, Taichung, 404 Taiwan; 8 Chinese Medicine Research Center, China Medical University, Taichung, Taiwan; 9 Department of Post-Baccalaureate Medicine, College of Medicine, National Chung Hsing University, Taichung, Taiwan

**Keywords:** Spinocerebellar ataxia type 3, Handgrip strength, Neurofilament light chain, Scale for the assessment and rating of ataxia, Body mass index

## Abstract

**Background:**

Spinocerebellar ataxia type 3 (SCA3) is an inherited, autosomal, and rare neurodegenerative disease. Serum/plasma biomarkers or functional magnetic resonance imaging used to assess progression, except for neurological examinations, is either inconvenient or expensive. Handgrip strength (HGS) may be considered as a biomarker to predict the progress of SCA3 and align with the alteration of plasma neurofilament light chain (NfL) and Scale for the Assessment and Rating of Ataxia (SARA).

**Methods:**

Patients with SCA3 and healthy subjects were recruited from Changhua Christian Hospital. SARA, body mass index (BMI), and NfL were obtained for both groups. HGS was measured using a Jamar Plus + hand dynamometer.

**Results:**

This study recruited 31 patients and 36 controls. HGS in the SCA3 group revealed a profound decrease (*P* < 0.001) compared with normal subjects. HGS also had a negative correlation with SARA (r =  − 0.548, *P* = 0.001), NfL (r =  − 0.359, *P* = 0.048), and a positive correlation with BMI (r = 0.680, *P* < 0.001). Moreover, HGS/BMI ratio correlated with SARA (r =  − 0.441, *P* = 0.013). Controlling for gender and age, HGS still correlated with the above clinical items. The initial hypothesis was also proved in SCA3 84Q transgenic mice, showing grip strength weakness compared to normal mice.

**Conclusions:**

HGS can be an alternative tool to assess the clinical severity of SCA3. Further research is needed to investigate the underlying mechanisms.

**Supplementary Information:**

The online version contains supplementary material available at 10.1186/s13023-023-02948-3.

## Background

Spinocerebellar ataxia (SCA) is an autosomal inherited disease and one of the rare neurodegenerative diseases. SCA type 3 (SCA3), also known as Machado-Joseph disease, caused by abnormal CAG trinucleotide expansion greater than 62 at the gene of *ataxin-3*, is the most frequent SCA in most countries [1]. Common symptoms include imbalance, incoordination, muscle rigidity or spasticity, dysarthria, diplopia, and depression [2]. Scientists have been searching for disease-modifying therapies in recent years, such as varenicline, valproic acid, or Trehalose, because SCA3 is challenging to treat [3]. SCA3 is a slowly progressive neurodegenerative disease; however, monitoring the change of clinical status by neurological scales in a short period would be challenging [4].

Physicians are on the lookout for assessment tools that are both accessible and efficient when it comes to neurological status assessment for ataxia. The Scale for the Assessment and Rating of Ataxia (SARA) is a clinical scale dedicated to detecting disease progression of cerebellar symptoms [5]. Morphometric magnetic resonance imaging (MRI) offers vital insights into structural changes, such as volumetric reduction in cerebellum, basal ganglia, brain stem, and diencephalon. These are instrumental in confirming and enhancing our understanding of this disease [6]. Functional MRI has revealed cerebellar/cortical dissociation pattern specific to SCA3 patients, as opposed to healthy controls. This finding shed light on the evaluation of potential functional connectivity within cerebral-cerebellar motor networks [7]. Neurofilament light chain (NfL) is an emerging blood biomarker to evaluate neuroaxonal damage such as Alzheimer’s disease, multiple sclerosis, postoperative delirium, multiple system atrophy, and amyotrophic lateral sclerosis [8–10]. NfL also correlates with the disease severity of SCA3 and predicts its pre-ataxic and ataxic stages [11, 12]. Urinal polyglutamine ataxin-3 protein is elevated in patients with SCA3 [13]. while polyglutamine ataxin-3 in plasma or peripheral blood mononuclear cells correlates with disease severity [14, 15]. However, fMRI and the above biomarkers, except for SARA, are either expensive or inconvenient. Developing an easy and affordable assessment tool is highly anticipated and much needed for clinical use.

Handgrip strength (HGS), which is measured using a portable hand-held dynamometer, has been a practical tool to assess the well-being of the elderly. HGS could predict longitudinal declines in cognition, functional status, and mortality in older community populations [16]. The United Kingdom Biobank data reported that higher dementia incidence and mortality were independently associated with lower grip strength [17]. Grip strength could also assess muscle activity, which predicts functional loss in patients with Parkinson’s disease, where striatal dopamine and impaired motor unit degeneration are recruited [18]. Proper grip strength cutoff points are good predictors of functional independence and wheelchair skills for males with spinal cord injuries [19]. HGS might relate functional capacity or motor performance to cerebellar disorders in a small pilot study, including three patients with degenerative cerebellar ataxia, without mentioning the genetic background [20]. For animal models related to neurodegeneration diseases, HGS can be identified as a physiologic biomarker to forecast the disease course [21–23]. In a transgenic marmoset model of SCA3 diseases, age-associated decrease of HGS were significantly more prominent in transgenic symptomatic marmosets compared to either wide-type or asymptomatic marmosets [24]. However, the relationship between HGS and the clinical status of patients with SCA3 remains unknown.

Herein, we aim to establish the validity of HGS inspection as a predictor of the disease progress in patients and mice with SCA3, and its application in ataxia clinic for the disease following.

## Result

### Demographic data

**Fig. 1:**
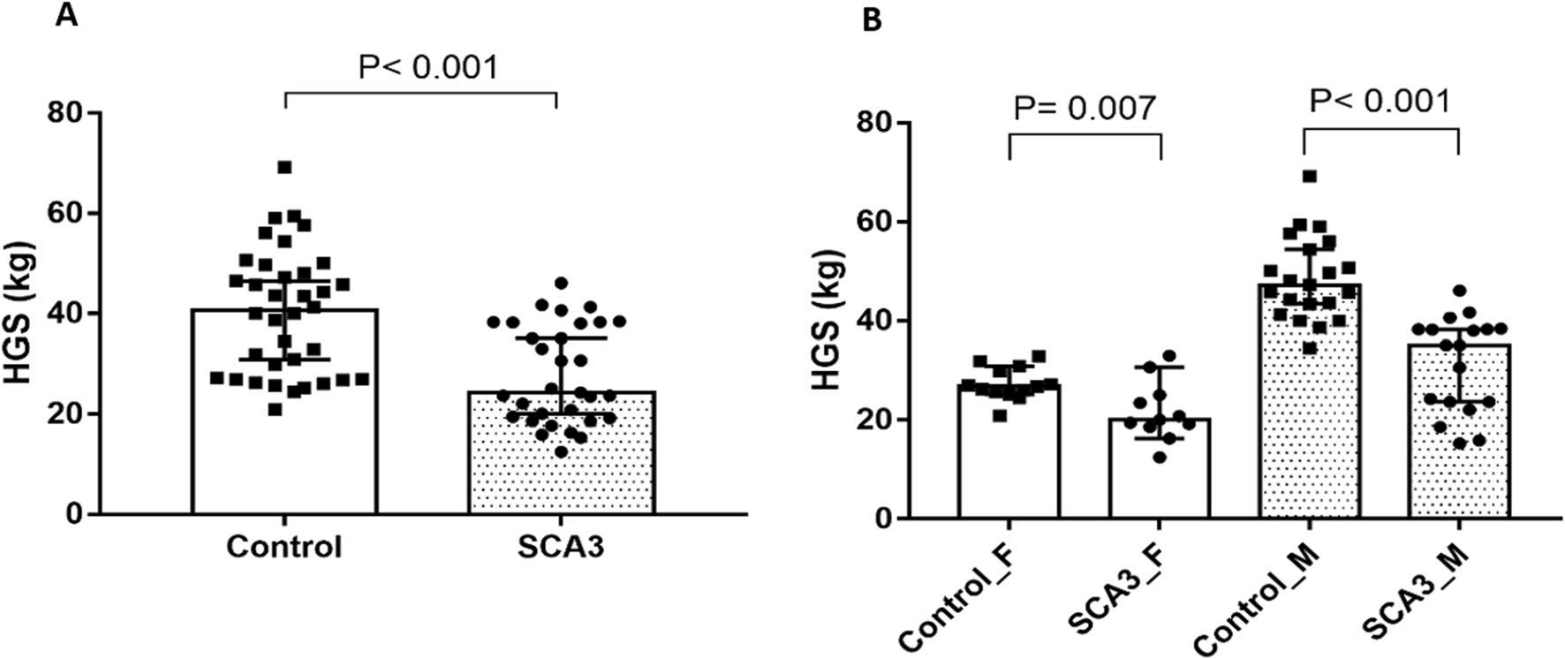
Handgrip strength of patients with SCA3 and healthy controls. Values are given as median with 95% coefficient interval (CI) for comparison of both groups (**A)** and both genders (**B**). *P*: by Mann–Whitney U test

This study enrolled 31 participants with SCA3 and 36 healthy controls. No significant difference was found in age or gender between the two groups (age: *P* = 0.517, gender: *P* = 0.988), as shown in detail in Table 1. The BMI of participants in the SCA3 group was lower than that of the control group (21.5 kg/m^2^ [19.5–25.5] vs. 23.6 kg/m^2^ [21.8–25.6], *P* = 0.043), consistent with the previous studies [25, 26]. The SCA3 group exhibited weaker HGS (24.3 kg [19.2–38.2] vs. 40.7 kg [27.1–49.3], *P* < 0.001, Fig. 1A) and lower HGS/BMI ratio compared to the control group (1.19 kg/(kg/m^2^) [0.95–1.55] vs. 1.38 kg/(kg/m^2^) [1.11–1.79], *P* < 0.001). A decrease in HGS was observed in either SCA3 female (20.10 kg [18.60–25.10] vs. 26.80 kg [25.45–30.4], *P* = 0.007) or SCA3 male (35.10 kg [23.30–38.33] vs. 47.20 kg [42.35–55.25], *P* < 0.001) patients compared with normal subjects in Fig. 1B. Additionally, a notable difference was observed in the significantly elevated plasma level of NfL in patients with SCA3 as compared to that in normal subjects (28.01 pg/mL [20.65–32.75] vs. 6.35 pg/mL [4.22–7.63], *P* < 0.001, Table 1). An elevated NfL level was found to predict the severity of SARA within the SCA3 group (r = 0.436, *P* = 0.014; see Additional file 1), consistent with previous reports [11, 12].

**Table 1:** Demographic data for patients with SCA3 and control subjects

	Control	SCA3	*P* value
Case number	36	31	
Male (%)	22 (61.1%)	19 (61.3%)	0.988
Age (years)	47.0 (32.0–53.5)	49.0 (40.0–55.0)	0.517
BMI (kg/m^2^)	23.6 (21.8–25.6)	21.5 (19.5–25.5)	0.043*
Handgrip strength (kg)	40.7(27.1–49.3)	24.3 (19.2–38.2)	< 0.001*
Handgrip strength (kg)/BMI (kg/m^2^)	1.38 (1.11–1.79)	1.19 (0.95–1.55)	< 0.001*
Disease status			
The age of onset (years old)	N/A	36 (32–45)	
Duration (years)	N/A	10 (5–12)	
CAG repeat count	N/A	71.0 (69.0–74.0)	
SARA	N/A	15.0 (8.5–21.0)	
Plasma			
NfL (pg/mL)	6.35 (4.22–7.63)	28.01 (20.65–32.75)	< 0.001*

Values are median with interquartile range

The intergroup comparison for gender was analyzed using the chi-square test, while the Mann–Whitney U test was used for other variables

*BMI* Body mass index, *SARA* Scale for the assessment of rating of ataxia, *NfL* Neurofilament light chain, N/A Not applicable, *P < 0.05

### Correlation between HGS and clinical items

**Fig. 2:**
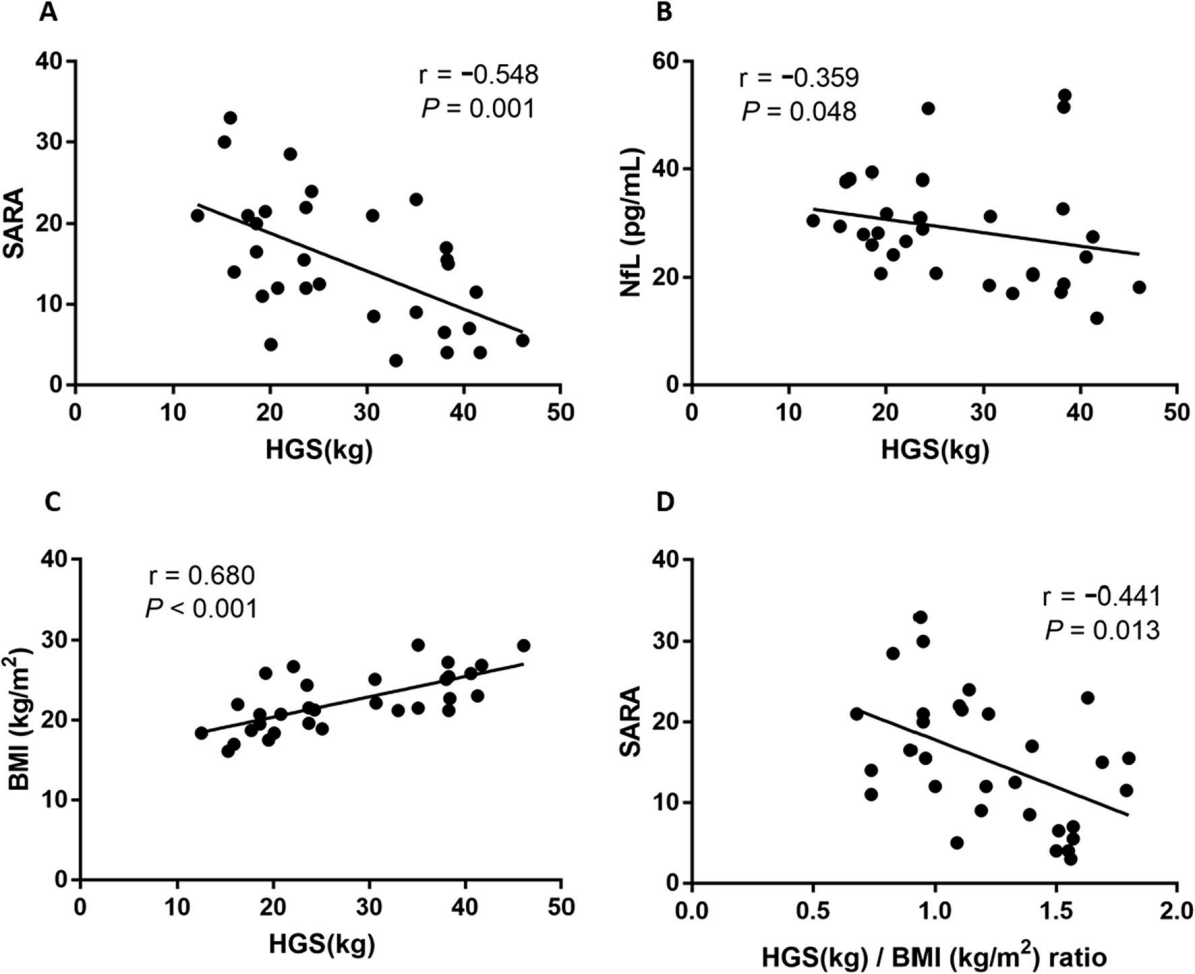
Correlation between HGS and SARA, NfL, and BMI (as shown in **A**–**C**). Correlation between HGS/BMI ratio and SARA (**D**). r: correlation coefficient, *P*: by Spearman rank test

The high strength of HGS can predict low scoring on SARA (r =  − 0.548, *P* = 0.001, Fig. 2A) and NfL (r =  − 0.359, *P* = 0.048, Fig. 2B) in SCA3 patients. Additionally, greater HGS was associated with lower scores in each subscale of SARA, including gait (r =  − 0.525, *P* = 0.002), stance ( r =  − 0.487, *P* = 0.005), sitting (r =  − 0.636, *P* < 0.001), speech disturbance (r =  − 0.522, *P* = 0.003), finger chase (r =  − 0.510, *P* = 0.003), finger-nose test (r =  − 0.355, *P* = 0.050), fast alternating hand movements (r =  − 0.505, *P* = 0.004), and heel-shin slide (r =  − 0.403, *P* = 0.025), as shown in Table 2.

**Table 2:** Correlation between HGS and eight subscales of SARA

Variable	Correlation coefficient	*P* value
*HGS*		
Gait	− 0.525	0.002
Stance	− 0.487	0.005
Sitting	− 0.636	< 0.001
Speech disturbance	− 0.522	0.003
Finger chase	− 0.510	0.003
Finger-nose test	− 0.355	0.050
Fast alternating hand movements	− 0.505	0.004
Heel-shin slide	− 0.403	0.025

Spearman rank test was used for correlation

*HGS* Handgrip strength, *SARA* Scale for the assessment and rating of ataxia

In the SCA3 patient group, HGS was effectively indicative of BMI (r = 0.680, *P* < 0.001, Fig. 2C). BMI has been demonstrated as a disease progression predictor in SCA3 [25, 26], and our study revealed a significant negative correlation with SARA (r =  − 0.420, *P* = 0.019; see Additional file 1). BMI has been correlated with HGS in most studies [27, 28]; thus, the HGS/BMI ratio was calculated as a new variable after adjusting HGS for BMI. The HGS/BMI ratio still exhibited a negative correlation with SARA scores (r =  − 0.441, *P* = 0.013, Fig. 2D). Even after considering gender and age as controlling factors, HGS continued to illustrate a consistent relationship with SARA, NfL, and BMI. Moreover, lower HGS can predict SCA3 patients with a higher CAG repeat number under adjusting for age (r =  − 0.396, *P* = 0.030; see Additional file 2).

### Grip strength in SCA3 mice

**Fig. 3:**
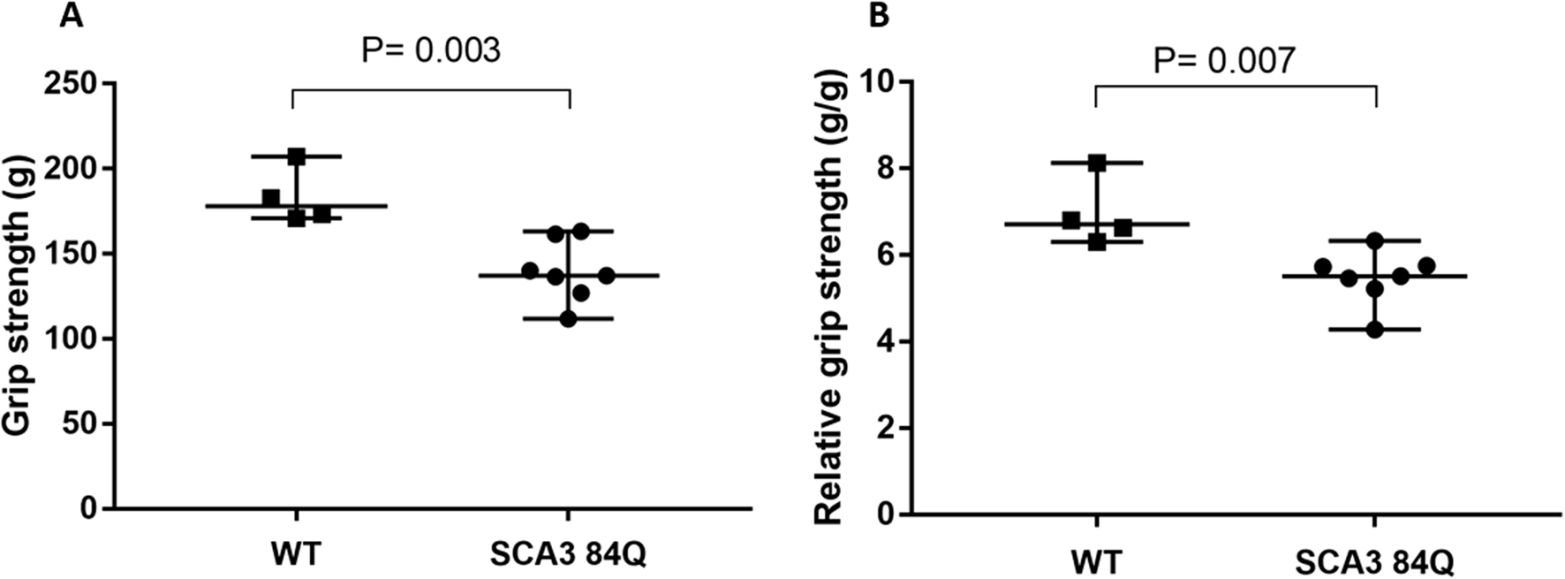
Four-limb grip strength (**A**) and relative grip strength (**B**) in micewith WTand transgenic SCA384Q mice. Data expressed as median with 95% CI (WT, n = 4; SCA3 84Q, n = 7). *P*: by Mann–Whitney U test

The grip strength of SCA3 84Q transgenic mice was significantly decreased at the age of 18 months than that of WT mice (*P* = 0.003, Fig. 3A). After correcting for body weight, the grip strength of SCA3 mice remained lower when compared to that of the WT mice(*P* = 0.007, Fig. 3B).

## Discussion

The current assessment of disease progression in SCA3 involves neurological examinations, such as SARA, for evaluating ataxia. NfL [12] and BMI [26] have established themselves as reliable predictors of SCA3, and our research uncovered a robust relationship between HGS and SARA, even after adjusting for gender and age. The increased strength of HGS can predict higher BMI and lower plasma levels of NfL. Additionally, a higher HGS/BMI ratio indicated lower scores in the SARA evaluation. Moreover, our study demonstrated that patients with SCA3 exhibited significantly lower HGS compared to healthy controls. This study represents the first instance where HGS or the HGS/BMI ratio has independently predicted the disease severity of SCA3, including all eight subscores of SARA. HGS can serve as an easily accessible tool to estimate the disease status of SCA3 in a clinical setting, even when considering the gene loading of SCA3-CAG repeat expansion.

HGS is a widely used measure of muscular strength, but various factors can influence its accuracy. HGS in patients with SCA3 may relate to myopathy, neuropathy or other comorbidity. Our study revealed a significant inverse correlation between HGS and disease severity, even after controlling for common factors. Various conditions, including arthritis, tendinitis, and major vascular or neurological disorders, can affect grip strength. Other factors, such as sex, age, handedness, and nutritional status, also impact HGS [29]. Participants with severe comorbidities, such as stroke, cancer, heart failure, or kidney failure, were excluded, and the absence of arthritis or tendinitis was screened to ensure the accuracy of our study. Handedness was determined according to the standard protocol for using a hand dynamometer [30], while sex and age were considered during statistical analysis.

Accordingly, recent literature revealed a report investigating grip strength in transgenic SCA3 135Q mice without mentioning the pathogenesis that contributes to the reduction of grip strength [31]. In the present study, we found a similar result in SCA3 84Q mice with weak grip strength compared with WT mice during the late disease stage. To account for the potential confounding effect of decreased body weight in SCA3 84Q mice at 18 months, we adjusted for body weight and still detected weaker grip strength in SCA3 mice compared to WT mice. Our previous studies have also demonstrated a decrease in the ratios of muscle mass and body weight in the quadriceps, gastrocnemius, tibialis, extensor digitorum longus, and soleus muscles compared to WT mice. The cross-sectional area of muscle fibers was found to be reduced in SCA3 84Q mice [32]. Atrophic signaling involving Akt/Forkhead box-O and myosin heavy chain (MyHC) expressions were implicated in these findings, indicating the existence of sarcopenia or muscle disease [33, 34]. Specifically, our study revealed a significant decrease in phosphorylated AKT and the muscle cell differentiation marker, MyHC, in SCA3 84Q mice compared to WT mice which indicated the possible muscular pathogenesis involved in the disease of SCA3 [32].

Muscle weakness is a common feature in patients with SCA3, and recent studies indicate that myopathic origin may contribute to distal muscle weakness in these individuals [35]. Further, evidence indicates a potential association between SCA3 and sarcopenia, as patients with SCA3 display lower muscle strength and lean mass than healthy controls [36]. Therefore, the muscle atrophy pathway may be related to the lower HGS observed in patients with SCA3 who have sarcopenia. Our study revealed that sarcopenia-related BMI and HGS could provide valuable insights into the clinical progression of patients with SCA3. A declining BMI signifies deterioration in the condition of patients with SCA3 and may indicate the possible occurrence of comorbidity with sarcopenia. Another report suggests that hyperkinesia may lead to increased energy expenditure, while dysphagia results in decreased nutrition intake, both potentially contributing to a body composition resembling sarcopenia [36]. The Foundation for the National Institutes of Health Biomarkers Consortium Sarcopenia Project also recommends grip strength measurements, such as HGS and HGS/BMI ratio, as non-Dual-energy X-ray absorptiometry approaches to identify sarcopenic patients among the elderly [37]. Our study revealed that HGS not only helps assess the clinical severity of SCA3 but is also significantly related to BMI. HGS can be regarded as a valuable tool for evaluating the progression of SCA3 patients with sarcopenia.

Therefore, further investigation into the possible mechanism of decreased HGS, including peripheral neuropathy, such as electromyography, nerve conduction studies, or even muscle biopsy, should be considered as the next step. In the future, we will conduct long-term longitudinal observations to examine the relationship between HGS and disease progression and expand our sample size to include more SCA3 patients and normal subjects.

## Conclusions

This is the first report on HGS in patients with SCA3. HGS exhibited an inverse correlation with the neurological status, namely SARA and NfL, and demonstrated a strong correlation with BMI, which is another predictor of disease progression. The increased HGS/BMI ratio consistently showed a relationship with SARA, providing valuable insights into disease severity. Combined with the grip strength information obtained from SCA3 84Q mice in the present study, HGS can serve as a practical and accessible tool to evaluate the clinical status of SCA3.

## Methods

### Patient recruitment

Subjects diagnosed with SCA3 by genetic mutational screening were recruited at Changhua Christian Hospital (CCH), Taiwan. The inclusion criteria for patients were a SARA score of ≥ 3 points [38]. Informed consent was collected from May 2021 to January 2022 (CCH-IRB approval No:200703). Participants were aged from 20 to 80 years. Exclusion criteria were pregnancy, comorbidity with stroke, cancer, heart failure, or renal failure. Patients with < 3 Points on the SARA scale were excluded. Demographic data such as age, age of disease onset, disease duration, CAG trinucleotide repeat number, and body mass index (BMI) were recorded. Age- and sex-matched individuals with negative genetic screening for mutant *ataxin-3* were recruited as healthy controls from the normal population of staff in CCH (CCH-IRB approval No:200730).

### HGS

A single physician measured the HGS with a Jamar Plus + hand dynamometer (Sammons Preston Rolyan, Bolingbrook, IL, USA). Participants were asked to sit on a straight back chair, keep their elbow flexed at 90 degrees, and hold the dynamometer with handle position 2, which is the distance of 4.8 cm from the handle to the fixed part of the dynamometer [30]. Participants had to apply maximal power for 3 s by the dominant hand. Grip strength was measured three times, with rest for 15 s between each effort [39]. The handgrip strength was the maximal value of the three measurements.

### SARA

A single experienced neurologist measured SARA for the SCA3 group. SARA is a universal scale to assess the severity of ataxia. SARA has eight subscores, including gait, stance, sitting, speech disturbance, finger chase, nose-finger test, fast alternating hand movements, and heel-shin slide [40]. The total scores ranged from 0 to 40, with higher points indicating worse cerebellar conditions.

### Plasma NfL

Blood was collected in BD EDTA tubes and then centrifuged at 2500 × g for 10 min at 4 °C to obtain plasma for each participant. Plasma samples were diluted at a ratio of 1:4. The single-molecule (Simoa) array technology by an ultra-high sensitivity protein molecular detection instrument (Simoa HD-X, Quanterix, MA, USA) and the Simoa NfL Advantage kit (Quanterix, MA, USA) measured the plasma NfL level [41]. All NfL values were within the linear ranges of the assays. The average intraassay coefficient of variation was 4.94%.

### Animal model and grip strength

C57BL/6 wild-type (WT) mice were obtained from the National Laboratory Animal Center (Taipei, Taiwan), and SCA3 84Q transgenic mice (C57BL/6 background) have been previously described [42]. The Institutional Animal Care and Use Committee of Changhua Christian Hospital approved all animal experiments (approval number: CCH-AE-108-021). The genotype of SCA3 84Q mice was confirmed through the polymerase chain reaction of a DNA sample obtained from the mouse tail (primer sequences for forward: 5′-TGGCCTTTCACATGGATGTGAA-3′, reverse: 5′-CCAGTGACTACTTTGATTCG-3′). The 430-bp molecule refers to the positive of SCA3 84Q. Mice received standard diets and were housed under a 12-h light/dark cycle in a temperature-controlled room. The body weights of the mice were recorded weekly to monitor their health status. Grip strength tests were performed only on 18-month-old mice. A Digital Force Gauge (Model DPS-5R: range of 0–5 kgf, Imada, Japan) was used to measure the mice’s four-limb grip strength. The mouse was placed on a metal grid, and its tail was gently pulled back in parallel, and the apparatus automatically recorded the peak force when the mouse released its grip. The maximal force (grams) was represented as four-limb grip strength, while relative grip strength was normalized to the weight of the mouse. Each mouse underwent a grip strength test three times at 1-min intervals [43].

### Statistics

Continuous variables were presented as the medians and interquartile ranges (25th–75th percentile), whereas the categorical variables were presented as numbers and percentages because most of the continuous variables did not follow a normal distribution. The Mann–Whitney U test was used to assess the difference in continuous variables between the healthy and diseased populations, while the chi-square test was employed for categorical variables. Additionally, the Spearman rank test was used to determine the strength of the relationship between the two variables. The partial correlation was used to adjust for age and gender. All data were analyzed using IBM Statistical Package for the Social Sciences for Windows, Version 22.0 (IBM Corp., Armonk, NY). A *P* value of < 0.05 was considered statistically significant.

### Supplementary Information

**Additional file 1: Fig. S1.** C orrelation between NfL and SARA in the SCA3 group. Plasma NfL positively correlated with SARA. **Fig. S2.** Correlation between BMI and SARA in the SCA3 group. BMI negatively correlated with SARA.

**Additional file2 : Table 1. **Correlation between HGS and A ge, Gender, SARA, NfL, BMI and CAG repeat count.

## Data Availability

We understand the importance of providing a data availability statement as part of our research manuscript. While we acknowledge the significance of transparency and reproducibility, due to privacy and confidentiality concerns, we are unable to publicly disclose the data used in this study. We assure you that the data supporting our findings will be made available to interested researchers upon reasonable request, subject to ethical and legal considerations. For inquiries regarding access to the data, please contact Chin-San Liu (liu48111@gmail.com) and Chung-Min Chiu (shongdiah@gmail.com). We are committed to promoting scientific integrity and collaboration while maintaining the confidentiality of sensitive information.

## Abbreviations

SCA: Spinocerebellar ataxia
SCA3: SCA type 3
SARA: Scale for the assessment and rating of ataxia
MRI: Magnetic resonance imaging
NfL: Neurofilament light chain
HGS: Handgrip strength
CCH: Changhua Christian Hospital
BMI: Body mass index
WT: Wild-type
MyHC: Myosin heavy chain
