# Supplementary material for: A pilot study: handgrip as a predictor in the disease progression of SCA3
Source: Orphanet J Rare Dis. 2023 Oct 11;18:317. doi: 10.1186/s13023-023-02948-3 (PMC10565987; doi:10.1186/s13023-023-02948-3)
Supplement: Supplementary file 1 — Additional file 1: Fig. S1. C orrelation between NfL and SARA in the SCA3 group. Plasma NfL positively correlated with SARA. Fig. S2. Correlation between BMI and SARA in the SCA3 group. BMI negatively correlated with SARA. [file 13023_2023_2948_MOESM1_ESM.pdf]

Additional files

Figure Legend

Figure 1. Correlation between NfL and SARA in the SCA3 group. Plasma NfL positively correlated with SARA.

Figure 2. Correlation between BMI and SARA in the SCA3 group. BMI negatively correlated with SARA.

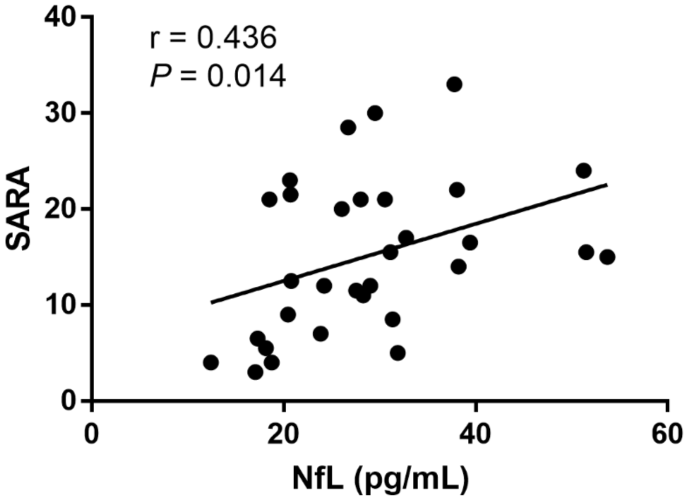

Figure 1

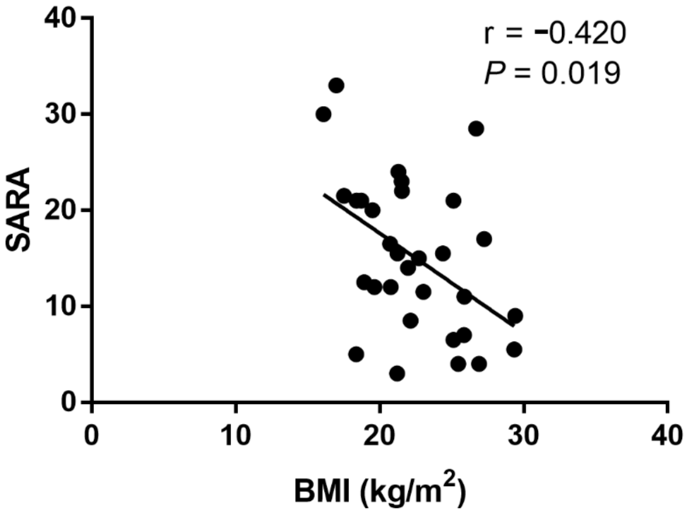

Figure 2
