# Supplementary material for: A pilot study: handgrip as a predictor in the disease progression of SCA3
Source: Orphanet J Rare Dis. 2023 Oct 11;18:317. doi: 10.1186/s13023-023-02948-3 (PMC10565987; doi:10.1186/s13023-023-02948-3)
Supplement: Supplementary file 2 — Additional file2 : Table 1. Correlation between HGS and A ge, Gender, SARA, NfL, BMI and CAG repeat count. [file 13023_2023_2948_MOESM2_ESM.pdf]

Additional file

TABLE 1. Correlation between HGS and Age, Gender, SARA, NfL, BMI and CAG repeat count

Variable	Correlation coefficient	*P*-value[&]
**HGS**		
Age	−0.131	0.484
Gender	0.507	0.004
SARA	−0.722	<0.001[#]
NfL	−0.418	0.024[#]
BMI	0.605	0.001[#]
CAG repeat count	−0.396	0.030[*]

[&]Spearman rank test was used for correlation

[#]Adjusted for age and gender

[*]Adjusted for age

HGS: Handgrip strength; SARA: scale for the assessment and rating of ataxia

NfL: Neurofilament light chain; BMI: Body Mass Index
